# Intermediate honeycomb ordering to trigger oxygen redox chemistry in layered battery electrode

**DOI:** 10.1038/ncomms11397

**Published:** 2016-04-18

**Authors:** Benoit Mortemard de Boisse, Guandong Liu, Jiangtao Ma, Shin-ichi Nishimura, Sai-Cheong Chung, Hisao Kiuchi, Yoshihisa Harada, Jun Kikkawa, Yoshio Kobayashi, Masashi Okubo, Atsuo Yamada

**Affiliations:** 1 Department of Chemical System Engineering, School of Engineering, The University of Tokyo, Hongo 7-3-1, Bunkyo-ku, Tokyo 113-8656, Japan; 2 Elements Strategy Initiative for Catalysts & Batteries (ESICB), Kyoto University, Nishikyo-ku, Kyoto 615-8245, Japan; 3 Department of Applied Chemistry, School of Engineering, The University of Tokyo, Bunkyo-ku, Tokyo 113-8656, Japan; 4 Institute for Solid State Physics, The University of Tokyo, Kashiwa, Chiba 277-8581, Japan; 5 Synchrotron Radiation Research Organization, The University of Tokyo, Tatsuno, Hyogo 679-5165, Japan; 6 Advanced Key Technologies Division, National Institute for Materials Science, Tsukuba, Ibaraki 305-0044, Japan; 7 Department of Engineering Science, The University of Electro-Communications, Chofu, Tokyo 182-8585, Japan; 8 RIKEN Nishina Center for Accelerator-Based Science, RIKEN, Wako, Saitame 351-0198, Japan

## Abstract

Sodium-ion batteries are attractive energy storage media owing to the abundance of sodium, but the low capacities of available cathode materials make them impractical. Sodium-excess metal oxides Na_2_MO_3_ (M: transition metal) are appealing cathode materials that may realize large capacities through additional oxygen redox reaction. However, the general strategies for enhancing the capacity of Na_2_MO_3_ are poorly established. Here using two polymorphs of Na_2_RuO_3_, we demonstrate the critical role of honeycomb-type cation ordering in Na_2_MO_3_. Ordered Na_2_RuO_3_ with honeycomb-ordered [Na_1/3_Ru_2/3_]O_2_ slabs delivers a capacity of 180 mAh g^−1^ (1.3-electron reaction), whereas disordered Na_2_RuO_3_ only delivers 135 mAh g^−1^ (1.0-electron reaction). We clarify that the large extra capacity of ordered Na_2_RuO_3_ is enabled by a spontaneously ordered intermediate Na_1_RuO_3_ phase with ilmenite O1 structure, which induces frontier orbital reorganization to trigger the oxygen redox reaction, unveiling a general requisite for the stable oxygen redox reaction in high-capacity Na_2_MO_3_ cathodes.

## Abstract (web summary)

Sodium-excess metal oxides Na_2_MO_3_ are appealing cathode materials for sodium-ion batteries. Here, the authors demonstrate that honeycomb-type cation ordering in Na_2_RuO_3_ triggers the oxygen redox reaction via frontier orbital reorganization, increasing the capacity by 1/3 compared with disordered Na_2_RuO_3_.

## Graphical abstract

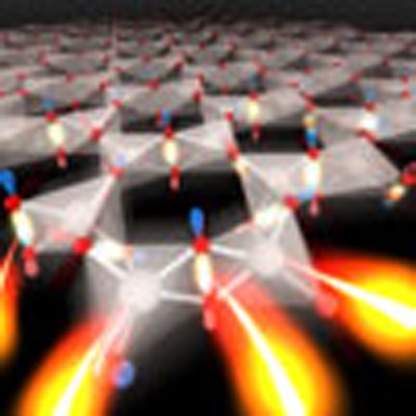

Li-ion batteries power almost all of today's portable electronics and their ability to store energy is increasingly important for large-scale applications such as electric vehicles and power grids. However, Li resources are limited and unevenly distributed geographically; hence, alternatives need to be found. Na-ion batteries have garnered great attention as candidates for large-scale applications owing to the abundance and low cost of sodium. Accordingly, Na-ion (de)intercalation compounds have been explored intensively^1^,^2^,^3^,^4^,^5^,^6^,^7^,^8^.

Among the various positive electrode materials that have been investigated, layered transition metal oxides NaMO_2_ (M=transition metal) are considered to be the most promising^3^,^9^,^10^,^11^,^12^,^13^, in part owing to their large theoretical capacities and the simple analogies from the successful application of LiMO_2_ in Li-ion battery systems. However, the stably cyclable capacity of NaMO_2_ is too small for practical use because deintercalation of a large amount of Na ions from the Na layer between the MO_2_ slabs usually causes irreversible structural changes^14^,^15^.

Replacing M atoms in the MO_2_ slab with Na (for example, Na[Na_1/3_M_2/3_]O_2_ or Na_2_MO_3_) is an appealing strategy to suppress these structural changes over a wider compositional range. The [Na_1/3_M_2/3_]O_2_ slabs supply additional Na ions, thereby increasing the reversible capacity while suppressing the over-deintercalation of Na ions from the Na layer. This ‘*A*_2−*x*_MO_3_' strategy has already been adopted in many lithium systems such as Li_2_MnO_3_–LiMO_2_ and Li_2_RuO_3_, in which enhanced capacities exceeding a M^4+^/M^3+^ one-electron reaction have been achieved through additional oxygen redox contribution^16^,^17^,^18^.

In our previous work, we applied this strategy to sodium systems, using a model compound, Na_2_RuO_3_ (Na[Na_1/3_Ru_2/3_]O_2_)^19^. However, despite the presence of [Na_1/3_Ru_2/3_]O_2_ slabs, Na_2_RuO_3_ delivered a modest capacity of 135 mAh g^−1^, limited to the Ru^5+^/Ru^4+^ one-electron reaction. In contrast, Rozier *et al.*^20^ very recently reported that Na_2_RuO_3_ can deliver a capacity greatly exceeding that of the Ru^5+^/Ru^4+^ one-electron reaction. At present, the discrepancy between the reported electrochemical properties is an open question. More generally, the requisite conditions for successful application of the ‘*A*_2−*x*_MO_3_' strategy for large-capacity sodium systems are poorly understood.

Here we demonstrate that a highly stabilized intermediate phase with honeycomb-type cation ordering in the [Na_1/3_M_2/3_]O_2_ slab is critical for the effective use of the ‘*A*_2−*x*_MO_3_' strategy, by comparing two polymorphs of Na_2_RuO_3_; namely, ‘ordered' Na_2_RuO_3_ with honeycomb-ordered [Na_1/3_M_2/3_]O_2_ slabs and ‘disordered' Na_2_RuO_3_ with randomly distributed [Na_1/3_M_2/3_]O_2_ slabs (Fig. 1).

## Results

### Structural characterization of Na_2_RuO_3_

**Figure 1:**
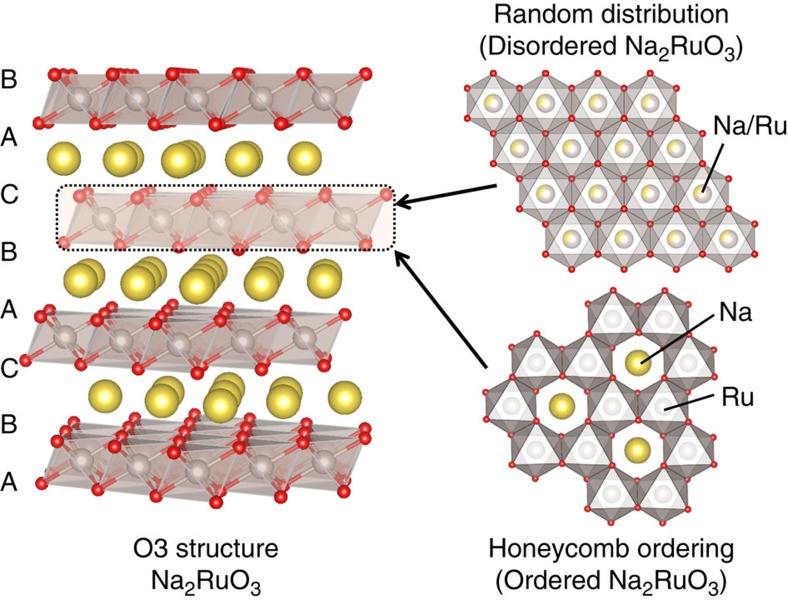
Structure of ordered and disordered Na_2_RuO_3_. Oxide ions (red) stack in the manner of ABCABC while both Na (yellow) and Ru (grey) occupy octahedral sites for both Na_2_RuO_3_. Ordered Na_2_RuO_3_ has the honeycomb-type cation ordering in the [Na_1/3_Ru_2/3_]O_2_ slab. Disordered Na_2_RuO_3_ has the randomly distributed [Na_1/3_Ru_2/3_]O_2_ slab.

**Figure 2:**
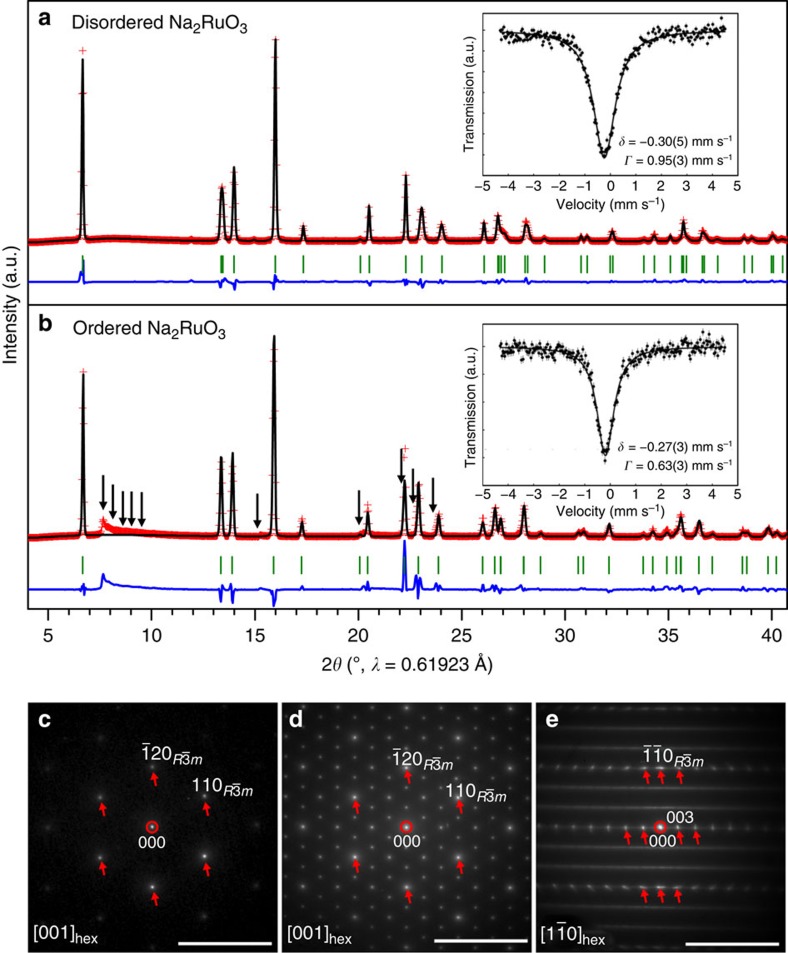
Structural characterization of disordered and ordered Na_2_RuO_3_. Observed and calculated (Rietveld method) synchrotron X-ray diffraction patterns for (**a**) disordered and (**b**) ordered Na_2_RuO_3_. Red crosses: experimental, black line: calculated, blue line: difference and green bars: Bragg positions. The black arrows in **b** indicate the superstructure peaks that were not considered for the refinement. Insets of **a**,**b** correspond to the ^99^Ru Mössbauer spectra recorded at 4.2 K for both pristine materials. Vertical error bars represent 1*σ* s.d. of counting statistics. Selected area electron diffraction (SAED) patterns in the (**c**) [001]_hex_ zone axes of disordered Na_2_RuO_3_, and in the (**d**) [001]_hex_ and (**e**) [1
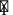
0]_hex_ zone axes of ordered Na_2_RuO_3_. The red circles and arrows, respectively, indicate the central and fundamental diffraction spots, which are common to disordered Na_2_RuO_3_. Un-marked diffraction spots in between correspond to superstructure peaks. Scale bars, 10 nm^−1^.

Ordered Na_2_RuO_3_ (hereafter denoted O-Na_2_RuO_3_) was synthesized by a thermal decomposition method, in which Na_2_RuO_4_ was annealed at 850 °C for 48 h under an Ar atmosphere^21^. Disordered Na_2_RuO_3_ (hereafter denoted D-Na_2_RuO_3_) was synthesized for comparison according to our previously reported procedure^19^. The ^99^Ru Mössbauer spectra of both compounds (insets in Fig. 2a,b) show a singlet absorption peak with an isomer shift around −0.3 mm s^−1^, which is a typical value for Ru^4+^ (for example, −0.25 mm s^−1^ for Y_2_Ru_2_O_7_ and −0.33 mm s^−1^ for SrRuO_3_)^22^, suggesting successful formation of stoichiometric Na_2_RuO_3_ compositions from both syntheses. The synchrotron X-ray diffraction (XRD) pattern of D-Na_2_RuO_3_ (Fig. 2a) is fully indexed to the O3 structure (*R*
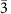
*m* space group, *a*=3.0969(3) Å and *c*=15.970(2) Å); all diffraction peaks are well-fitted by Rietveld refinement with a structural model in which Na and Ru are randomly distributed in the [Na_1/3_Ru_2/3_]O_2_ slabs (Fig. 1 and Supplementary Table 1). Na in the Na layer occupies octahedral sites and the oxide ions are stacked in an ABCABC arrangement (O3 structure)^23^. The selected area electron diffraction (SAED) pattern along the [001]_hex_ zone axis (Fig. 2c) shows diffraction spots fully indexed by the *R*
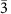
*m* model with disordered [Na_1/3_Ru_2/3_]O_2_ slabs.

The synchrotron XRD pattern of O-Na_2_RuO_3_ (Fig. 2b and Supplementary Table 1) is similar to that of D-Na_2_RuO_3_ and is almost fitted by Rietveld refinement in the *R*
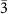
*m* space group (*a*=3.1195(5) Å and *c*=15.989(4) Å) assuming disordered [Na_1/3_Ru_2/3_]O_2_ slabs. However, diffuse scatterings remain, as highlighted by the black arrows in Fig. 2b. Similar characteristics have been reported for many *A*_2_MO_3_ (*A*=Li, Na and M=transition metal(s)) materials, and usually arise from honeycomb ordering in the MO_2_ slabs^16^,^17^,^20^. Indeed, the SAED pattern along the [001]_hex_ zone axis (Fig. 2d) shows many extra diffraction spots, which are not predicted using the standard *R*
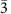
*m* model with disordered [Na_1/3_Ru_2/3_]O_2_ slabs. These spots can be indexed to a 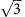
*a*_hex_ × 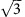
*b*_hex_ supercell arising from the honeycomb ordering of Na and Ru in the [Na_1/3_Ru_2/3_]O_2_ slabs (Fig. 1). In contrast, the SAED pattern along the [1
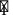
0]_hex_ zone axis (Fig. 2e) shows extra streaks along [001]_hex_. This feature is characteristic of stacking faults in honeycomb-ordered [*A*_1/3_M_2/3_]O_2_ slabs along the *c*_hex_ direction, explaining the diffusive nature of the superstructure reflections in Fig. 2b (ref. 24).

### Electrochemical properties of Na_2_RuO_3_

**Figure 3:**
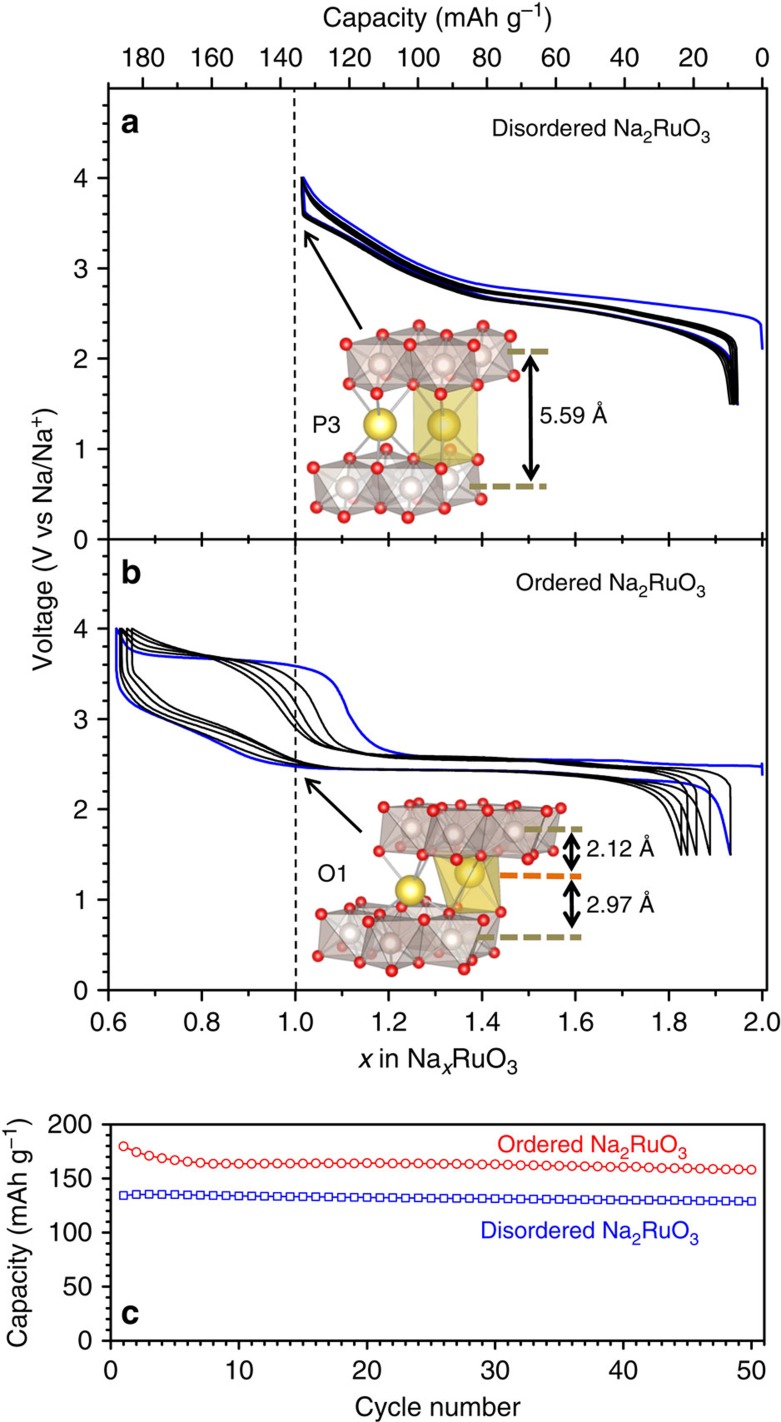
Electrochemical properties of disordered and ordered Na_2_RuO_3_. Galvanostatic cycling curves recorded at 30 mA g^−1^ for (**a**) disordered and (**b**) ordered Na_2_RuO_3_ with the first cycle highlighted in blue. Insets show the coordination environment of Na at *x*=1.0 for each phase. (**c**) Capacity retentions for (blue squares) disordered and (red circles) ordered Na_2_RuO_3_.

Having demonstrated the differences between O- and D-Na_2_RuO_3_ in terms of the in-plane ordering and stacking sequences of the [Na_1/3_Ru_2/3_]O_2_ slabs, the Na^+^ (de)intercalation properties of both materials were studied to clarify the influence of the honeycomb ordering. Figure 3a,b shows the charge–discharge curves measured between 1.5 and 4.0 V versus Na/Na^+^ at 30 mA g^−1^ (a rate of ∼C/5). Here charging is an anodic process (Na deintercalation) and discharging is a cathodic process (Na intercalation). D-Na_2_RuO_3_ (Fig. 3a) delivers a reversible capacity of 135 mAh g^−1^, corresponding to (de)intercalation of 1.0 Na^+^, which is consistent with our previous report^19^. In contrast, the reversible capacity of O-Na_2_RuO_3_ exceeds 180 mAh g^−1^, indicating reversible (de)intercalation of 1.3 Na^+^, beyond a Ru^5+^/Ru^4+^ one-electron redox process. This behaviour is in agreement with that of Na_2_RuO_3_ reported by Rozier *et al.*^20^. Importantly, the voltage profile of O-Na_2_RuO_3_ significantly differs from the S-shaped voltage profile of D-Na_2_RuO_3_. O-Na_*x*_RuO_3_ (0.7≤*x*≤2) exhibits a staircase-like charge profile with a first voltage plateau around 2.5 V for 1.0≤*x*≤2.0 and a second voltage plateau around 3.6 V for 0.7≤*x*≤1.0; the second voltage plateau is related to the extra capacity of O-Na_2_RuO_3_, which exceeds that of the Ru^5+^/Ru^4+^ one-electron reaction. Although the plateau at 3.6 V shows gradual narrowing with repeating the cycles presumably due to slight loss of the crystallinity (Supplementary Fig. 1a,b), O-Na_2_RuO_3_ shows excellent capacity retention of 160 mAh g^−1^ after 50 cycles (Fig. 3c), which indicates the remarkable stability of the redox processes that contribute to the increased capacity. Indeed, the voltage plateau around 3.6 V is clearly observed for every charge process, suggesting occurrence of the accumulative oxygen redox reaction (Supplementary Fig. 1c,d)^18^. Therefore, the available capacity significantly exceeds one-electron redox reaction for O-Na_2_RuO_3_ even after 50 cycles. It is surprising that the in-plane honeycomb-type cation ordering in O-Na_2_RuO_3_ is solely responsible for the drastic changes in the electrochemical properties that results in 30% higher capacity. To the best of our knowledge, this is the first demonstration of the critical role of honeycomb-type cation ordering in [*A*_1/3_M_2/3_]O_2_ slabs in determining the primary electrochemical properties.

### Structural change during (de)sodiation

**Figure 4:**
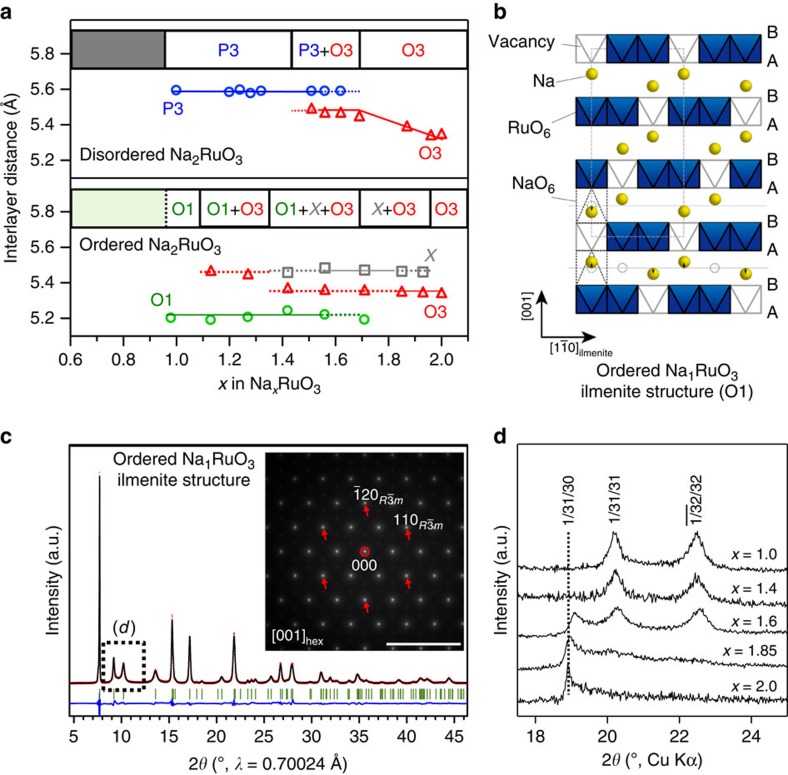
Structural changes of disordered and ordered Na_2_RuO_3_ during charge. (**a**) Interlayer distance of the different phases involved on desodiation as a function of *x* in disordered and ordered Na_*x*_RuO_3_. (**b**) Crystal structure of ilmenite-type Na_1_RuO_3_. All Na ions displace cooperatively towards Na vacancies in the honeycomb planes. (**c**) Observed and calculated (Rietveld method) synchrotron X-ray diffraction patterns for ordered Na_1_RuO_3_. Red crosses: experimental, black line: calculated, blue line: difference and green bars: Bragg positions. The inset shows the selected area electron diffraction (SAED) pattern for ilmenite-type Na_1_RuO_3_. Scale bar, 10 nm^−1^ (**d**) *Ex situ* XRD patterns showing the evolution of the superstructure peaks for ordered Na_*x*_RuO_3_ on charge.

To clarify the reaction mechanisms in Na_2_RuO_3_, we studied the structural changes of D-Na_2_RuO_3_ and O-Na_2_RuO_3_ during cycling (Fig. 4a–d). As reported previously^19^ and further supported by *ex situ* XRD patterns (Supplementary Fig. 2), D-Na_*x*_RuO_3_ undergoes a structural change from O3 to P3 (with Na in prismatic sites owing to the ABBCCA oxide-ion stacking) on charging^23^. This O3→P3 transition is commonly observed in O3-NaMO_2_ materials through gliding of the [Na_1/3_Ru_2/3_]O_2_ slabs from ABCABC (O3) to ABBCCA (P3) stacking^9^,^25^. The synchrotron XRD pattern of the charged state (D-Na_1_RuO_3_; Supplementary Fig. 3 and Supplementary Table 2) is fully fitted by the Rietveld refinement assuming a P3 structure (*R*
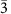
*m* space group, *a*=2.927(2) Å and *c*=16.774(12) Å), in which Na ions in the Na layer are located in prismatic sites (inset in Fig. 3a). The interlayer distance is significantly increased from 5.323(1) Å (*x*=2) to 5.591(4) Å (*x*=1) because of the high aspect ratio of the prismatic Na sites in the P3 structure (Fig. 4a).

It should be emphasized that O-Na_2_RuO_3_ exhibits a completely different structural evolution from D-Na_2_RuO_3_ on charging. The *in situ* and *ex situ* XRD patterns of O-Na_2_RuO_3_ (Supplementary Figs 4 and 5) at the first plateau show that an intermediate phase ‘X' with an unknown structure (presumably a P-type phase based on the interlayer distance of around 5.5 Å) appears during the initial stages of the desodiation, and that the O3 phase is eventually transformed to a new phase at *x*=1 with an extremely short interlayer distance of around 5.2 Å (Fig. 4a). These complex equilibria among the O3, X and O1 phases are responsible for the long voltage plateau observed in the 1≤*x*≤2 composition range of O-Na_*x*_RuO_3_. On charging, the diffuse scatterings of the superstructure gradually disappear, while new, well-resolved peaks appear (Fig. 4d). The well-resolved character of the new superstructure reflections suggest that the disordered stacking of the honeycomb lattice is spontaneously adjusted towards the ordered state during electrochemical desodiation. The Rietveld refinement and SAED pattern of O-Na_1_RuO_3_ (Fig. 4b,c and Supplementary Tables 2 and 3) confirm that the [Na_1/3_Ru_2/3_]O_2_ layers maintain the original honeycomb-type in-plane ordering. As shown in Fig. 4b, the ABCABC stacking sequence of oxygen is transformed to an ABAB sequence, in which Na in the Na layer occupies octahedral sites (O1 structure; inset in Fig. 3b). This ordered structure is isomorphic with the ilmenite FeTiO_3_-type structure^26^.

**Figure 5:**
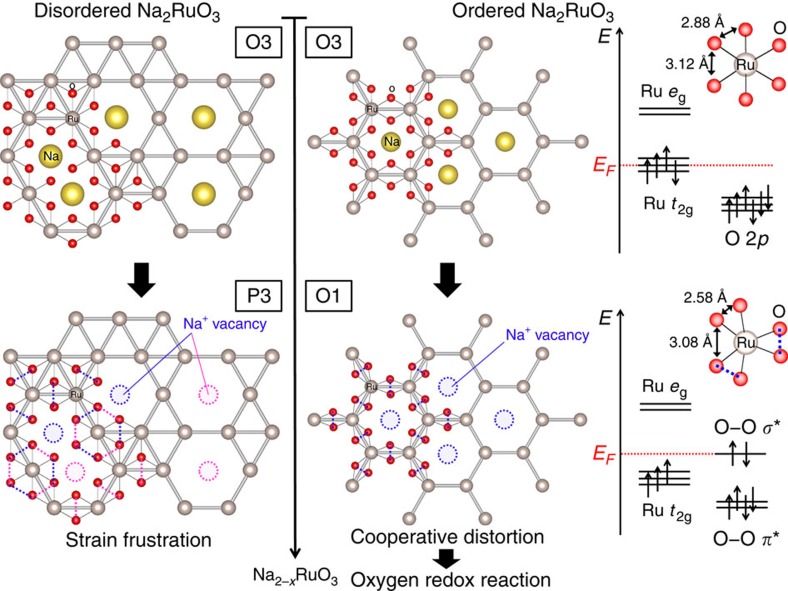
Reaction mechanisms of disordered and ordered Na_2_RuO_3_. Schematic representation of the structural changes during charge–discharge for disordered Na_2_RuO_3_ and ordered Na_2_RuO_3_. Ordered Na_2_RuO_3_ can distort cooperatively to raise the energy level of the antibonding σ* orbital of the O–O bond, leading to the oxygen redox reaction. Disordered Na_1_RuO_3_ cannot accommodate the RuO_6_ distortion due to strain frustration, which prevents the oxygen redox reaction.

In the refined structure of O-Na_1_RuO_3_ (*R*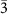 space group, *a*=5.2492(1) Å and *c*=15.6201(6) Å), the Na site in the [Na_1/3_Ru_2/3_]O_2_ slabs is vacant, which means Na was extracted from the [Na_1/3_Ru_2/3_]O_2_ slabs prior to the Na layers. In this very unique, stable intermediate ilmenite-type Na_1_RuO_3_, all NaO_6_ octahedra in the Na layer share faces with a RuO_6_ octahedron and a ‘□O_6_' octahedron of the adjacent [□_1/3_Ru_2/3_]O_2_ slabs (□: Na vacancy). As shown in Fig. 4a, the interlayer distance of O-Na_1_RuO_3_ (5.2067(2) Å) is much shorter than that of D-Na_1_RuO_3_ (5.591(4) Å) as a result of substantial displacement of Na in the Na layer towards □O_6_ octahedra by Coulombic attraction (Fig. 4b). Importantly, the RuO_6_ octahedron is strongly distorted: the shortest neighbouring O–O distance is 2.580(4) Å, whereas the longest is 3.080(6) Å (Fig. 5 and Supplementary Table 3).

The ilmenite structure of O-Na_1_RuO_3_ is stabilized by the following mechanisms: (1) the ordered Na arrangement minimizes Na^+^–Na^+^ repulsion; (2) the Na vacancy □ in the [□_1/3_Ru_2/3_]O_2_ slab strongly attracts Na ions in the face-sharing octahedra in adjacent Na layers; (3) the ordered and displaced arrangement of Na ions minimizes Ru^5+^–Na^+^ repulsive interactions; and (4) the cooperative distortion of the RuO_6_ octahedra can minimize the total strain energy in the ordered honeycomb lattice. All these mechanisms lead to the formation of the ilmenite phase with well-ordered stacking of the honeycomb lattices.

On discharge, *in situ* XRD patterns (Supplementary Fig. 4) prove that the phase transformation is almost reversible except for a small asymmetry, that is, the appearance of the X phase only at charge. Presumably, this parasitic asymmetric behaviour is observed under non-equilibrium conditions such as the microscale heterogeneity.

### Electronic structure of Na_2−*x*_RuO_3_

**Figure 6:**
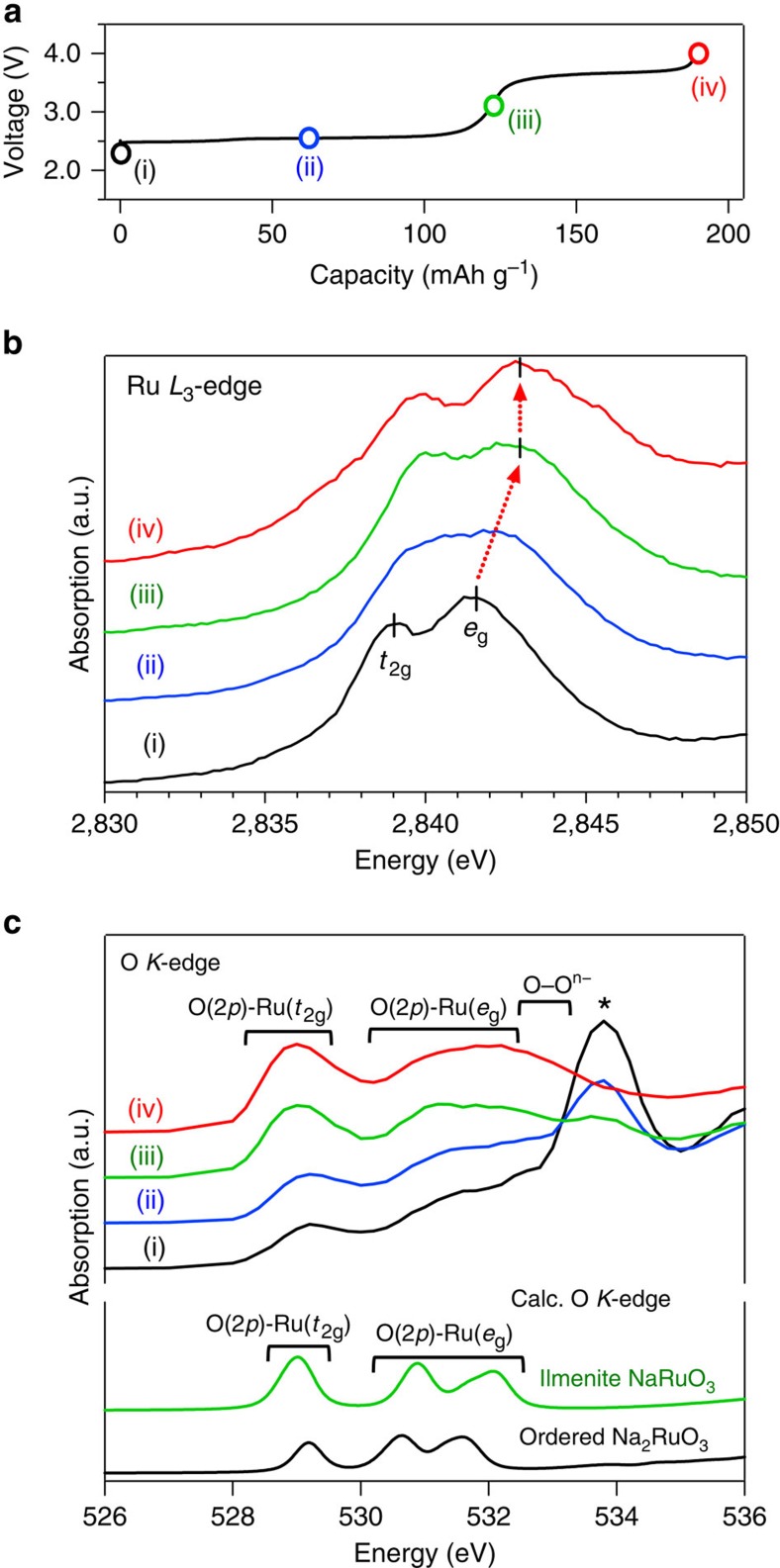
Electronic structure changes of ordered Na_2_RuO_3_ upon charge. (**a**) States of charge of the samples for X-ray absorption spectroscopy. (**b**) Ruthenium *L*_3_-edge and (**c**) oxygen *K*-edge X-ray absorption spectra for various O-Na_*x*_RuO_3_ compositions: (i) *x*=2.0 (black), (ii) *x*=1.5 (blue), (iii) *x*=1.0 (green) and (iv) *x*=0.62 (red). The asterisked peak corresponds to O 2*p*-Na 3*p*. The calculated oxygen *K*-edge spectra for ordered Na_2_RuO_3_ (black) and ilmenite-type NaRuO_3_ (green) are also shown.

After the O3→O1 transition, O-Na_2_RuO_3_ delivers additional capacity, exceeding that of the Ru^5+^/Ru^4+^ one-electron reaction at the higher voltage plateau of around 3.7 V. To clarify the overall redox mechanism in O-Na_2_RuO_3_, we conducted Ru *L*_3_-edge and oxygen *K*-edge X-ray absorption spectroscopy (XAS) in the partial fluorescence yield mode at various charge depths (Fig. 6, Supplementary Figs 6 and 7). The Ru *L*_3_-edge XAS directly probes the 4*d* orbitals through the Laporte-allowed 2*p*→4*d* transition^27^. Furthermore, because the O 2*p* orbital strongly hybridizes with the Ru 4*d* orbital, oxygen *K*-edge XAS can be used to monitor the hole created on the O 2*p* and Ru 4*d* orbitals on charging^28^. Note that the probing depth of the partial fluorescence yield mode is about 100 nm; hence, the spectra are bulk sensitive^29^.

The Ru *L*_3_-edge spectrum for pristine O-Na_2_RuO_3_ (*t*_2g_^4^*e*_g_^0^, neglecting the trigonal distortion of Ru for clarity), Fig. 6b, shows two absorption peaks corresponding to the excitation from the 2*p* orbital to the unoccupied *t*_2g_ and *e*_g_ orbitals. The peak position for the 2*p*→*e*_g_ absorption (2841.5 eV) is similar to that reported for Ru^4+^ (2841.6 eV) in SrRuO_3_ and RuO_2_ (ref. 27), confirming Ru^4+^ in pristine O-Na_2_RuO_3_. On charging to ilmenite-type Na_1_RuO_3_, two absorption peaks shift to higher energy. Since the peak position of the 2*p*→*e*_g_ absorption for ilmenite-type Na_1_RuO_3_ (2,843 eV) agrees with that of Ru^5+^ in Sr_4_Ru_2_O_9_ (ref. 27), oxidation from Ru^4+^ to Ru^5+^ occurs at the first potential plateau. However, further desodiation at the second plateau does not shift the 2*p*→*e*_g_ absorption peak, suggesting no substantial change in the valence state of Ru.

**Figure 7:**
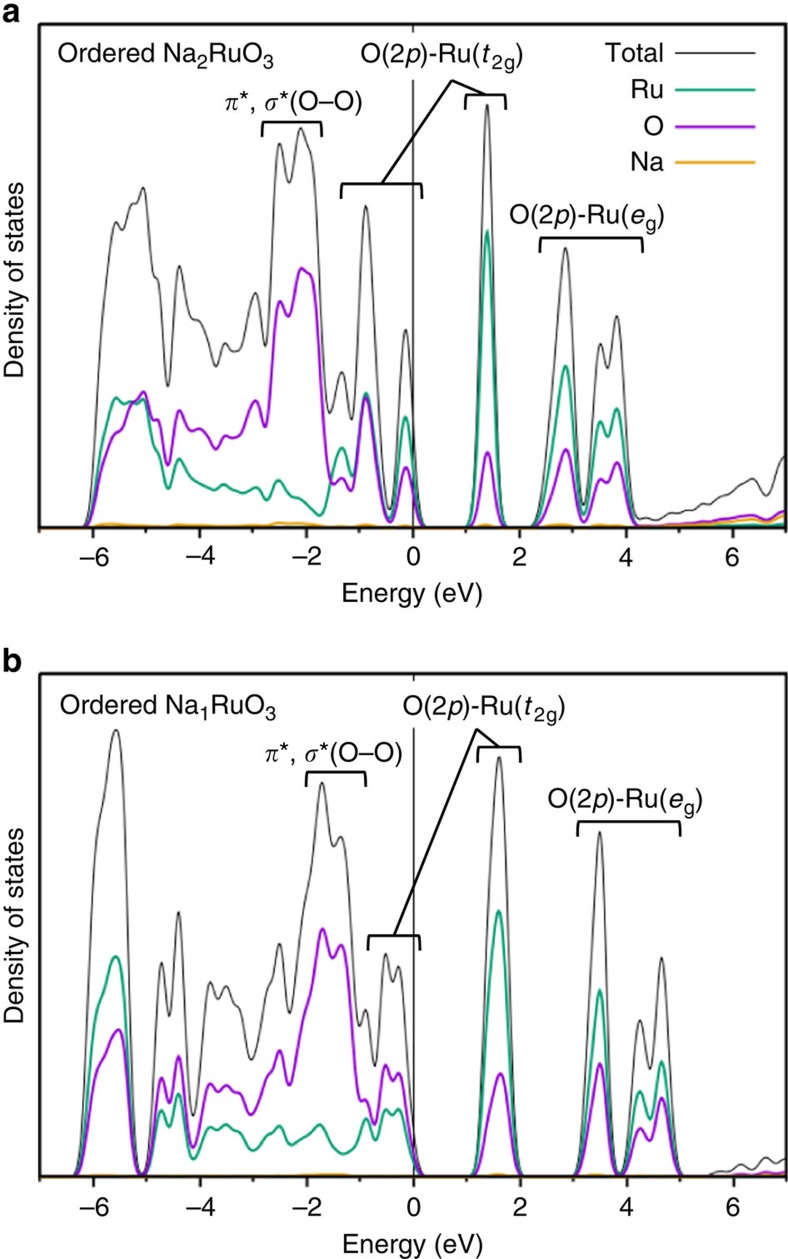
Calculated electronic structure of ordered Na_2_RuO_3_ upon charge. Calculated density of states (DOS) for ordered (**a**) O3-Na_2_RuO_3_ and (**b**) ilmenite-type Na_1_RuO_3_.

The O *K*-edge spectrum of pristine O-Na_2_RuO_3_ (*t*_2g_^4^*e*_g_^0^) shows a large peak at 534 eV, which is ascribed to the unoccupied hybridized orbital of O 2*p*–Na 3*p*, and its peak intensity simply reflects the amount of Na in the lattice^30^. The calculated density of states for O-Na_2_RuO_3_ (Fig. 7a) also indicates that the O 2*p*–Na 3*p* hybridized orbital exists in this energy range. For the lower energy region that represents the redox reaction, the calculated oxygen *K*-edge spectrum agrees well with the spectra from 528 to 533 eV. Thus, the peaks around 529 eV and 532 eV can be ascribed to the unoccupied hybridized orbitals of O 2*p*–Ru *t*_2g_ and O 2*p*–Ru *e*_g_, respectively^31^,^32^. At the voltage plateau of 2.5 V, desodiation diminishes the O 2*p*–Na 3*p* signal, whereas the peak intensity of O 2*p*–Ru *t*_2g_ (around 529 eV) increases on charging, indicating hole generation on the O 2*p*–Ru *t*_2g_ orbital. The increase in the peak intensity around 529 eV is also in good agreement with the calculated oxygen *K*-edge spectrum for ilmenite-type Na_1_RuO_3_.

On further charging at the second plateau (around 3.6 V), the peak intensity of the O 2*p*–Ru *t*_2g_ is almost constant, whereas the peak around 532 eV broadens as a result of the new absorption band emerging around 533 eV. This spectral change cannot be explained by Ru^5+^→Ru^6+^ oxidation (hole generation on O 2*p*–Ru *t*_2g_). One possible explanation is the formation of a new chemical bond involving oxygen. For example, formation of an O–O bond (that is, peroxo/superoxo-like groups O_2_^*n*−^ (1≤*n*≤3)) has been proposed as the oxygen redox mechanism for many Li_2_(M,M*′*)O_3_ cathode materials (M,M*′*=Fe, Ru, Sb, Sn or Ir)^18^,^33^,^34^. Here the hole generated by further charging is stabilized on the antibonding σ* orbital of the O–O bond which is covalently bonded to the transition metal. It should be noted that the ^99^Ru Mössbauer spectrum of O–Na_0.62_RuO_3_ (Supplementary Fig. 8) shows absorption with an isomer shift of *δ*=+0.21 mm s^−1^, which is a typical value for Ru^5+^ (+0.11 mm s^−1^ for Na_3_RuO_4_ and +0.19 mm s^−1^ for Ca_2_EuRuO_6_)^22^, supporting that no further Ru^5+^→Ru^6+^ oxidation occurs.

On discharge, while the 2*p*→*t*_2g_ and 2*p*→*e*_g_ signals in the Ru *L*_3_-edge spectra shift to the initial lower energy region, the O–O bond and O 2*p*–Ru *t*_2g_ signals in the O *K*-edge spectra decrease reversibly (Supplementary Figs 6 and 7). Furthermore, although the diffraction peaks of the *ex situ* XRD pattern after cycling exhibit weak broadening due to the slight loss of the crystallinity, O-Na_2_RuO_3_ still shows characteristic diffuse scatterings similar to the pristine compound (Supplementary Fig. 1b), which suggests that the honeycomb-type in-plane ordering is maintained during the charge–discharge cycles for all Na compositions, where 0.7≤*x*≤2. Importantly, this is the first simultaneous achievement of extra oxygen redox capacity and structural integrity leading to totally reversible oxygen participation. All other *A*_2−*x*_MO_3_-type electrodes suffer from severe structural rearrangement on the first charge and from lowering of the operating voltage during subsequent cycles^16^,^17^,^34^,^35^,^36^. This structural rearrangement occurs because M migrates from octahedral sites in the M layer to face-sharing tetrahedral sites in the adjacent *A* layer^35^. Presumably, this inter-site migration of Ru is suppressed in Na_2_RuO_3_ because of incompatibility of large Ru with the tetrahedral site. Furthermore, the interlayer O–O bond formation, which is predicted theoretically for delithiated Li_2_MnO_3_ to destabilize the oxygen redox reaction^37^, should be inhibited by the large interlayer distance in Na_*x*_RuO_3_ (for example, 5.2−5.5 Å for Na_*x*_RuO_3_ and 4.7−4.8 Å for Li_*x*_Ru_0.75_Mn_0.25_O_3_)^36^. As shown in Supplementary Fig. 9, the initial Coulombic efficiency of O-Na_2_RuO_3_ is higher (95%) than that of other *A*_2−*x*_MO_3_ (for example, 70% for Li_2_MnO_3_ and 90% for Li_2_RuO_3_)^17^,^36^,^38^,^39^.

## Discussion

The critical role of honeycomb ordering in Na_2_RuO_3_ based on the aforementioned original observations is summarized in Fig. 5. The ordered [□_1/3_Ru_2/3_]O_2_ slabs in ilmenite O-Na_1_RuO_3_ accommodate the distortion of the RuO_6_ octahedra cooperatively, whereby the shortest O–O distance is 2.580(4) Å. Because the short O–O distance raises the energy level of the antibonding σ* orbital of the O–O bond to the Fermi level, the oxygen redox reaction is triggered. Indeed, the calculated density of states for ilmenite O-Na_1_RuO_3_ (Fig. 7b) shows that the occupied antibonding orbitals of the O–O bond exist near the Fermi level. The naturally derived scenario is that, once the hole is generated on the antibonding σ* orbital of the O–O bond, the O–O distance becomes shorter^40^. This short distance leads to the higher energy level of the antibonding σ* orbital of the oxygen–oxygen bond and an acceleration of the oxygen redox reaction. This hypothesis was recently reinforced by McCalla *et al.*^34^: scanning transmission electron microscopy and neutron diffraction on fully delithiated Li_2_IrO_3_ (Li_0.5_IrO_3_) determined the shorter O–O distance (2.45 Å) after the oxygen oxidation. However, as demonstrated in this work, the short O–O distance is required before the oxygen oxidation to achieve the extra capacity. For example, the disordered [Na_1/3_Ru_2/3_]O_2_ slabs in D-Na_1_RuO_3_ cannot accommodate the cooperative distortion of the RuO_6_ octahedra owing to strain frustration. Thus, the O–O distances in D-Na_1_RuO_3_ are long enough (2.751(6) and 2.927(1) Å) so that the energy level of the antibonding σ* orbital of the O–O bond is well below the Fermi level, which inhibits the extra oxygen redox process within the electrolyte stability window. Therefore, honeycomb-type cation ordering in the [Na_1/3_M_2/3_]O_2_ slab is a structural requisite condition to trigger the stable oxygen redox capacity in Na_2_MO_3_.

Finally, we mention the role of Ru in the oxygen redox reaction. Very recently, Saubanère *et al.*^37^ theoretically demonstrated that the covalent bond between the peroxo-like (O_2_)^*n*−^ 2*p* and Ru 4*d* orbitals in Li_2_RuO_3_ (reductive coupling mechanism) is essential to facilitate the oxygen redox reaction and to suppress the oxygen gas release, whereas the M(3d)-O(2*p*) overlap is too small to enable this mechanism. For O-Na_2_RuO_3_, in addition to the stable structural nature inherent in the Na system by suppressing both the inter-site Ru migration and the interlayer O–O bond formation as discussed above, the similar reductive coupling mechanism through the large orbital overlap between the Ru 4*d* and O 2*p* orbitals should suppress the oxygen gas release, which explains the high cycle stability of O-Na_2_RuO_3_ compared with that of any other Li excess materials (Supplementary Fig. 9).

In conclusion, the present work demonstrates the critical role of honeycomb-type cation ordering in Na_2_MO_3_ to achieve enhanced reversible capacity based on the oxygen redox process. In comparison to its disordered polymorph, O-Na_2_RuO_3_ with in-plane honeycomb ordering exhibits a significantly different voltage profile, leading to a 30% extra capacity, spurred by the electrochemically driven, further ordered intermediate, NaRuO_3_, which accommodates cooperative distortion of the RuO_6_ octahedra. The short O–O distance in distorted RuO_6_ induces frontier orbital reorganization, triggering the oxygen redox reaction.

To the best of our knowledge, this is the first report that clarifies the underlying structural requirement to trigger the oxygen redox reaction in *A*_2−*x*_MO_3_. By further exploiting the compositional versatility of *A*_2_MO_3_ while meeting this requirement, there is great potential to develop oxygen redox electrodes for superior batteries. Considering the impact of the stable intermediate on the overall electrode properties, the peculiar phenomena and concepts demonstrated here may give general implications for a deeper understanding and improved utilization of intercalation materials.

## Methods

### Synthesis of Na_2_RuO_3_

Ordered Na_2_RuO_3_ was synthesized by the thermal decomposition method according to the literature where Na_2_RuO_4_ was annealed at 850 °C for 48 h under Ar atmosphere^21^. Disordered Na_2_RuO_3_ was synthesized according to the procedure we previously reported^19^. The NaHCO_3_ and RuO_2_ precursors were calcined at 850 °C for 12 h under Ar atmosphere. Both compounds were handled in a globe box filled with Ar (dew point below −110 °C).

### Materials characterization

Powder X-ray diffraction patterns were recorded on a Rigaku TTR-III diffractometer equipped with Cu *Kα* radiation in 0.02° steps over the 2*θ* range of 10–80°. XRD patterns were measured at SPring-8 BL02B2 and Photon Factory BL-8B. The samples were handled without air exposure throughout the experiments. Rietveld refinements were carried out using Jana2006 (ref. 41) or TOPAS-Academic software. The profile of Na_1_RuO_3_ was better described using independent crystallite size for 00*l* reflections, Popa's sphere harmonics for *h*0*l* and *hkl* reflections and different crystallite sizes for (*h*0*k*)=3*n* as implemented in TOPAS in order to take into account the stacking faults affecting part of the diffraction peaks. The crystal structures were drawn using VESTA^42^. SAED patterns were recorded using an electron microscope (HF-3000S; Hitachi Ltd.) operated at 300 kV and an imaging plate system (FDL-5000; Fujifilm). The camera length was calibrated with a Si crystal. ^99^Ru Mössbauer spectroscopy was carried out with the ^99^Rh source (*T*_1/2_=15 days), which was prepared by the ^99^Ru(p,n)^99^Rh reaction at the RIKEN AVF cyclotron. The ^99^Ru(p,n)^99^Rh reaction was conducted with a ^99^Ru (95% enriched) target under 12 MeV proton irradiation. Both the source and the samples were cooled in liquid He during the measurements. The samples were transferred into the liquid He cryostat without air exposure. The Doppler velocity was calibrated by measuring a ^57^Fe Mossbauer spectrum of α-Fe, and the zero velocity was determined by the isomer shift of Ru metal at 4.2 K.

### Electrochemical measurement

For the electrochemical measurements, 80 wt% of Na_2_RuO_3_ was mixed with 10 wt% of acetylene black and 10 wt% of polyvinylidene in *N*-methyl pyrrolidone to make a slurry, which was then coated onto Al foil. The dried films were used as a cathode in the 2,032-type coin cells with Na metal as an anode. NaPF_6_ (1 M) in ethylene carbonate/diethyl carbonate (1:1 v/v) was used as an electrolyte. The charge–discharge experiments were conducted at 30 mA g^−1^ between 1.5−4.0 V versus Na/Na^+^, within the electrolyte stability window.

### Samples for *ex situ* characterization

The electrodes for *ex situ* XRD, XAS and ^99^Ru Mossbauer spectroscopy were prepared as described above. O-Na_1_RuO_3_ samples for synchrotron XRD and TEM studies were prepared following the same method except that the positive electrode consisted in a pellet (Ø 10 mm, 50 mg) of O-Na_2_RuO_3_ that was sintered at 850 °C for 12 h. The batteries were then cycled to a designated voltage that was maintained for a few hours (Na_1_RuO_3_) or until a given current has been applied. After cycling, the batteries were introduced into an Ar-filled glovebox and the collected electrodes were washed three to five times with anhydrous dimethyl carbonate.

### *In situ* XRD

*In situ* XRD (*operando* mode) was carried out using an *in situ* cell purchased from Rigaku on a D8 ADVANCE powder diffractometer equipped with Co *Kα* radiation in 0.02° steps over the 2*θ* range of 17−25° (1.37 s per step, one pattern recorded every ≈12 min). The electrochemical part of the cell is similar to the one described above. The only exception lies in the preparation of the electrode whose slurry was coated on plastic films to be peeled off after drying. The current rate was C/20 (full (dis)charge theoretically achieved in 20 h), allowing us to record an XRD pattern every Δ*x*=0.02. Only selected patterns are shown in Supplementary Fig. 4 for clarity concerns. Electrochemistry was controlled and recorded using a Biologic VMP3.

### X-ray absorption

O *K*-edge XAS was carried out at BL07LSU of SPring-8. The samples were transferred to a vacuum chamber without air exposure. Bulk-sensitive partial-fluorescence yield (PFY) mode was used for the O *K*-edge XAS. Energy resolution was approximately 100 meV. All the spectra were recorded using a silicon drift detector at room temperature. The spectra were normalized by the peak intensity at 532 eV, because the peak at 532 eV corresponding to O 2*p*-Ru *e*_g_ may not be affected by the redox of Ru^5+^/Ru^4+^. Ru *L*_3_-edge XAS was carried out at BL27SU of SPring-8. The samples were transferred to a vacuum chamber without exposure to air. The spectra were recorded in the bulk-sensitive PFY mode using a silicon drift detector at room temperature.

### *Ab initio* calculation

Spin-polarized DFT+*U* calculations were performed using the VASP code^43^ with *U*_eff_ for Ru ranging from 0.5 to 4.5 eV. Van der Waals density functional optPBE^44^ was employed. The plane-wave energy cut-off was set to 520 eV and the Brillouin zone integration was done in a k-point grid chosen to converge the energy to 10^−4^ eV. Atomic coordinates and lattice parameters were relaxed using conjugate gradient method until the maximum force on atoms is <2 × 10^−3^ eV Å^−1^. Initial geometry of Na_2_RuO_3_ in the *C*2/*m* symmetry was taken from the literature^45^ while the initial geometry for NaRuO_3_ in the *R*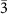 symmetry is from the present work. Antiferromagnetic ordering of spin on Ru was assumed, the pattern was set to be Néel type. The oxygen K-edge X-ray absorption pattern was calculated by the WIEN2k code^46^ employing the full potential linear augmented plane-wave method, dipole approximation to the golden rule for the transition probability was assumed.

## Additional information

**How to cite this article:** Mortemard de Boisse, B. *et al.* Intermediate honeycomb ordering to trigger oxygen redox chemistry in layered battery electrode. *Nat. Commun.* 7:11397 doi: 10.1038/ncomms11397 (2016).

## Supplementary Material

Supplementary Information Supplementary Figures 1-9 and Supplementary Tables 1-3.
