# Supplementary material for: Intermediate honeycomb ordering to trigger oxygen redox chemistry in layered battery electrode
Source: Nat Commun. 2016 Apr 18;7:11397. doi: 10.1038/ncomms11397 (PMC4837481; doi:10.1038/ncomms11397)
Supplement: Supplementary Information — Supplementary Figures 1-9 and Supplementary Tables 1-3. [file ncomms11397-s1.pdf]

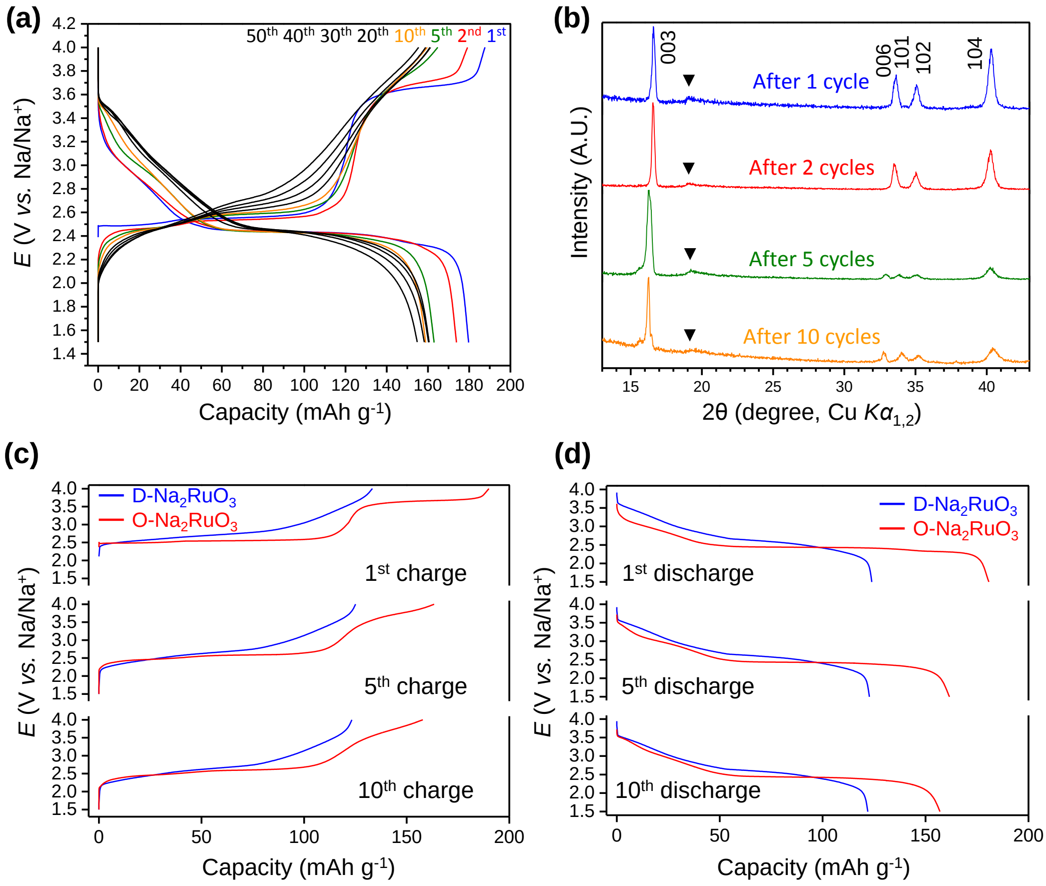

Supplementary Figure 1. (a) Evolution of the charge/discharge profiles of O-Na_2RuO_3 after the 1st, 2nd, 5th, 10th, 20th, 30th, 40th and 50th cycles. (b) XRD patterns of the electrodes at 1.5 V *vs.* Na^+/Na after the 1st, 2nd, 5th and 10th cycles. With repeating the cycle, the diffraction peaks become broad, suggesting slight decrease of the crystallinity. However, the arrowheads highlight that the superstructure peaks corresponding to the honeycomb lattice are still observed even after 10 cycles. (c) Comparison of the 1st, 5th, and 10th charge curves of D-Na_2RuO_3 and O-Na_2RuO_3. (d) Comparison of the 1st, 5th, and 10th discharge curves of D-Na_2RuO_3 and O-Na_2RuO_3. The charge curve shows a voltage plateau around 3.6 V in every cycle.

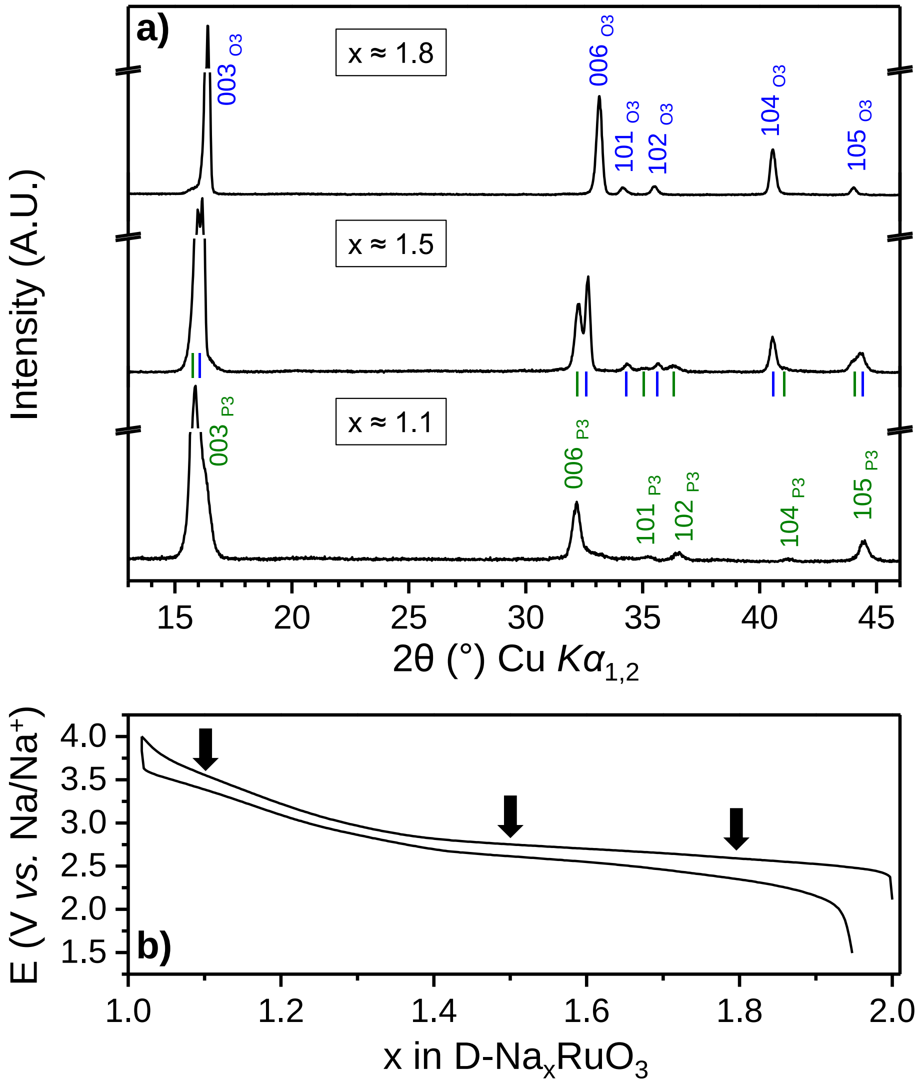

Supplementary Figure 2. (a) *Ex situ* XRD patterns of D-Na$_x$RuO$_3$ electrodes at x = 1.8, 1.5 and 1.2 upon charge. The *hkl* indices of the O3 (blue) and P3 (green) phases are given. (b) Charge-discharge curves of D-Na$_x$RuO$_3$. Arrows correspond to the Na contents given above.

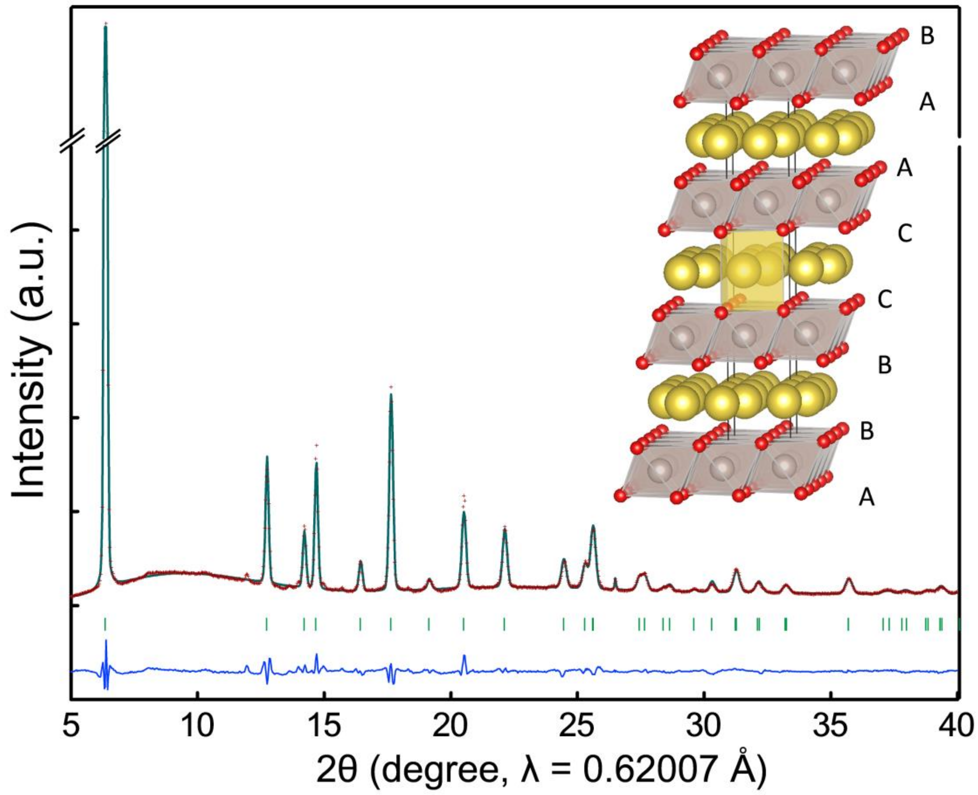

Supplementary Figure 3. Observed and calculated (Rietveld method) synchrotron X-ray diffraction patterns for disordered Na_1RuO_3. Red crosses: experimental, black line: calculated, blue line: difference and green bars: Bragg positions. The inset shows the resulting P3 structure.

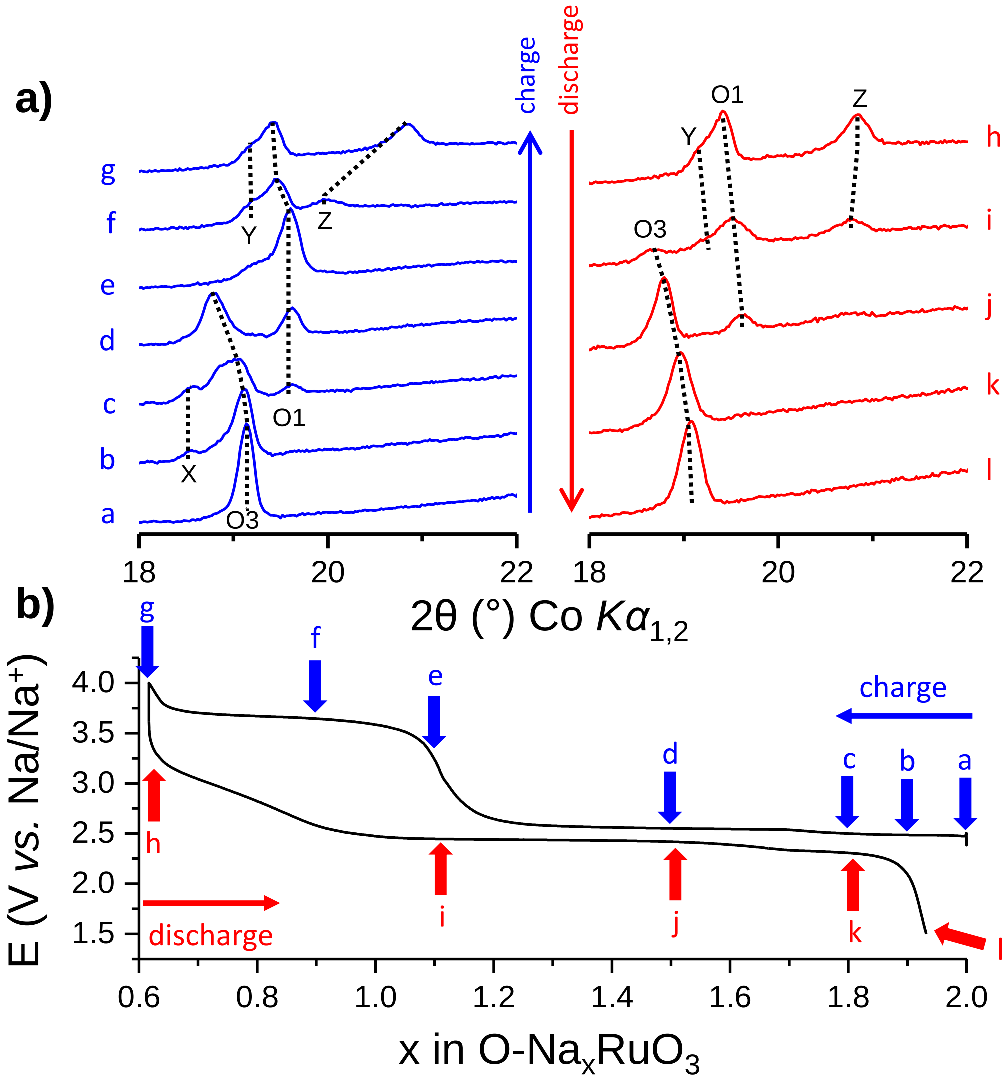

Supplementary Figure 4. (a) Selected *in situ* X-ray diffraction patterns recorded during the first cycle of a battery assembled with O-Na_2RuO_3 as positive electrode material. (b) The charge-discharge curve of O-Na_2RuO_3. The a-l labels as well as the blue and red arrows correspond to the different states of (dis)charge for the recorded diffraction patterns. While showing the overall structural reversibility, unidentified X phase is observed at the plateau around 2.7 V during the charge, which is similar to the *ex situ* XRD experiment. However, during the discharge, the X phase is not observed. Presumably, the X phase is formed kinetically as a metastable state during the charge.

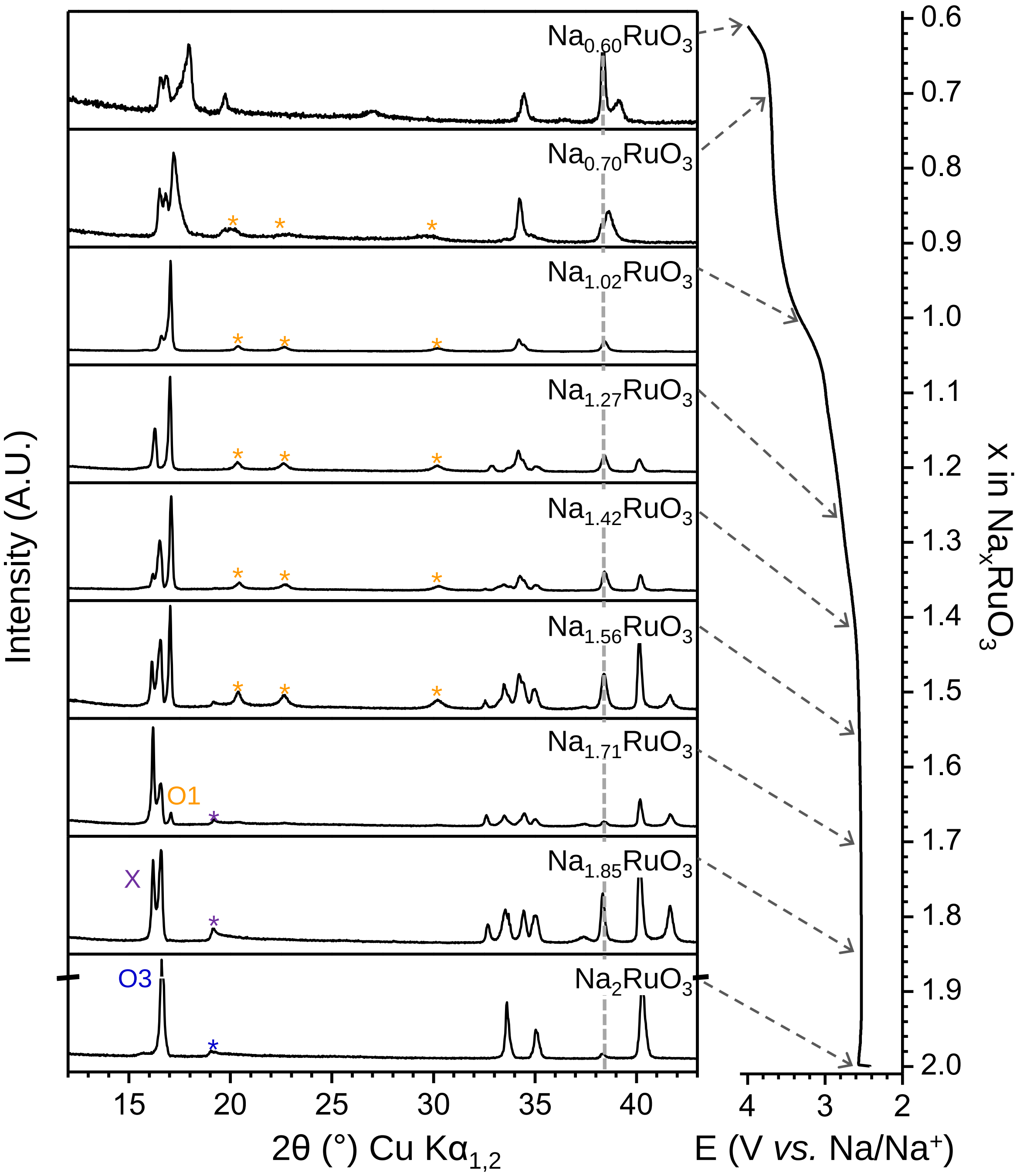

Supplementary Figure 5. Selected *ex situ* X-ray diffraction patterns recorded at different states of charge of O-Na_xRuO_3. The * symbols indicate the position of the superstructure peaks associated to (blue) Na_2RuO_3, (purple) X-Na_xRuO_3 and (orange) Na_1RuO_3. The three phases (O3, X, and O1) are clearly observed from $x = 1.71$ to 1.42. The vertical dashed line indicate the peak from the XRD sample holder.

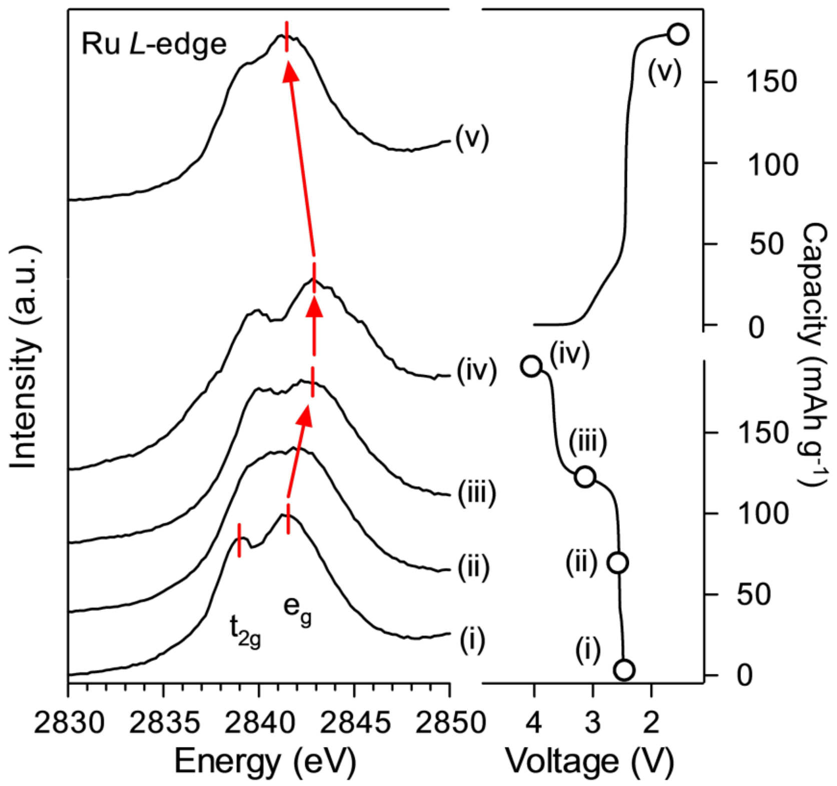

Supplementary Figure 6. *Ex situ* Ru L_3-edge X-ray absorption spectra during charge-discharge for ordered Na_xRuO_3. The (i)-(v) labels correspond to the state of (dis)charge highlighted on the galvanostatic curves.

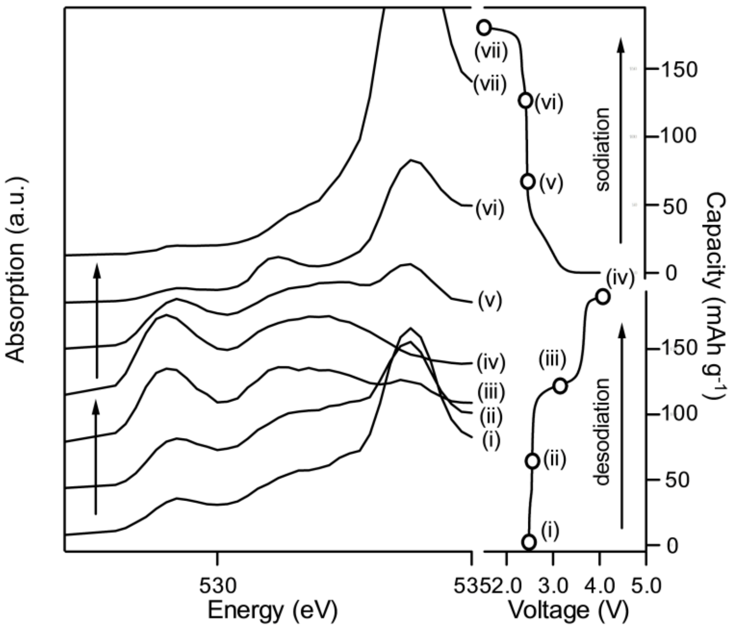

Supplementary Figure 7. *Ex situ* Oxygen *K*-edge X-ray absorption spectra during charge-discharge for ordered Na_xRuO_3. The (i)-(vii) labels correspond to the state of (dis)charge highlighted on the galvanostatic curves.

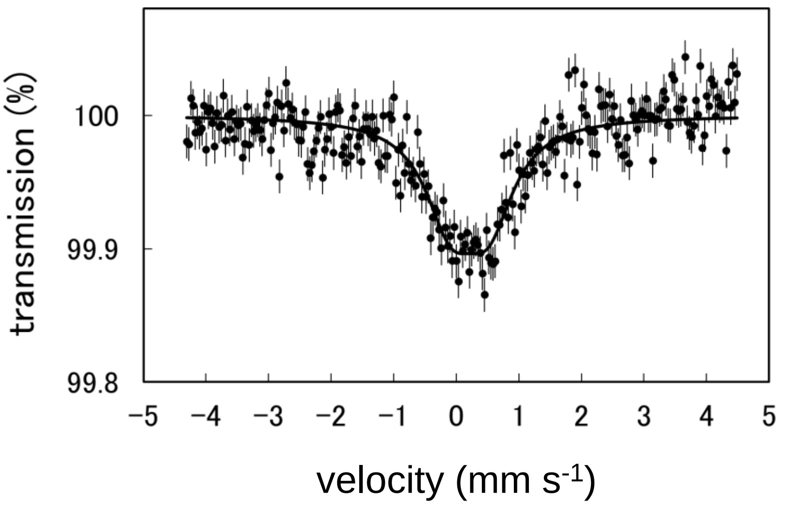

Supplementary Figure 8. ^{99}Ru Mössbauer spectrum of O-$Na_{0.62}RuO_3$ recorded at 4.2 K. Black dots: experimental, black line: fit with a doublet (δ = +0.21(6) mm s^{-1}, ΔE_Q = 0.60(5) mm s^{-1}, and Γ = 0.60(3) mm s^{-1}). Vertical error bars represent 1σ s. d. of counting statistics.

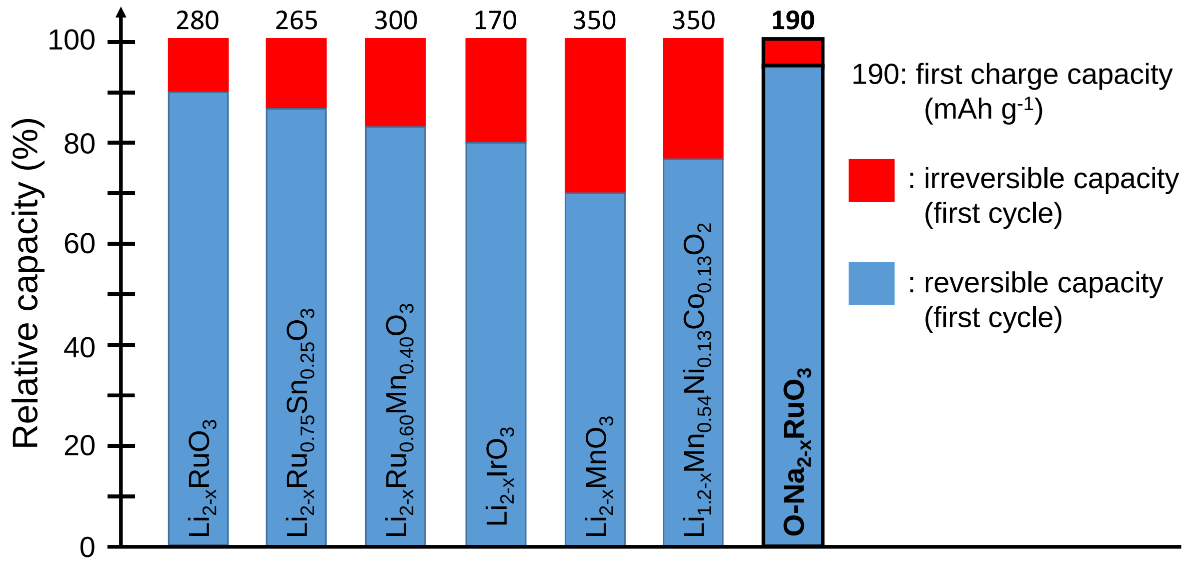

Supplementary Figure 9. Relative irreversible capacity of O-Na_2RuO_3 *vs.* that of various Li excess materials. Corresponding references can be found in the main text.

**a) Disordered Na_2RuO_3**						
Space group: $R\bar{3}m$:h, a = 3.0969(3) Å, c = 15.970(2) Å						
**Atom**	**Site**	**Fractional coordinates**			**Occupancy *g***	***B* (Å²)**
		x	***y***	***z***		
**Na(1)**	3*a*	0	0	0	0.333	0.62(4)
**Ru**	3*a*	0	0	0	0.667	
**Na(2)**	3*b*	0	0	1/2	1.0	2.1(2)
**O**	6*c*	0	0	0.267(1)	1.0	2.2(2)
R_{wp} = 8.50 %; R_B = 4.23 %; Berar's factor = 2.7.						

**b) Ordered Na_2RuO_3**						
Space group: $R\bar{3}m$:h, a = 3.1195(5) Å, c = 15.989(4) Å						
**Atom**	**Site**	**Fractional coordinates**			**Occupancy *g***	***B* (Å²)**
		x	***y***	***z***		
**Na(1)**	3*a*	0	0	0	0.333	0.10(7)
**Ru**	3*a*	0	0	0	0.667	
**Na(2)**	3*b*	0	0	1/2	1.0	1.0(3)
**O**	6*c*	0	0	0.263(1)	1.0	0.9(4)
R_{wp} = 11.79 %; R_B = 7.18 %; Berar's factor = 2.5 (with excluded superstructure peaks).						
R_{wp} = 16.52 %; R_B = 7.08 % (without excluded superstructure peaks).						

Supplementary Table 1. Structural parameters and reliability factors calculated from the synchrotron X-ray diffraction patterns for (a) disordered and (b) ordered Na_2RuO_3. The relatively high values of *B* for Na and O are explained by the weak stacking faults, which slightly distorts the local structure.

**a) Disordered Na_1RuO_3 ($Na_{0.708(12)}RuO_3$)**<br><br>Space group: $R\bar{3}m$:h, a = 2.927(2) Å, c = 16.774(12) Å						
**Atom**	**Site**	**Fractional coordinates**			**Occupancy *g***	***B*** **(Å²)**
		x	***y***	***z***		
**Na**	6*c*	0	0	0.1600(6)	0.237(4)	3.0
**Na(2)**	3*a*	0	0	0	0	-
**Ru**	3*a*	0	0	0	2/3	1.73(5)
**O**	6*c*	1/3	2/3	0.0647(3)	1	3.02(14)
R_{wp} = 5.41 %; R_p = 3.92% ; GoF = 2.93 ; R_B = 1.28 %						

**b) Ordered Na_1RuO_3**<br><br>Space group: $R\bar{3}$:h, a = 5.2492(1) Å, c = 15.6201(6) Å						
**Atom**	**Site**	**Fractional coordinates**			**Occupancy *g***	***B*** **(Å²)**
		x	***y***	***z***		
**Na**	6*c*	0	0	0.1413(3)	1	3.00(1)
**Ru**	6*c*	0	0	0.33623(8)	0.87(2)*	0.5
**Ru**	3*a*	0	0	0	0.13(2)*	0.5
**O**	18*f*	0.2811(8)	-0.0600(9)	0.2596(1)	1	1.0
R_{wp} = 9.47%; R_p = 6.97% ; GoF = 2.54 ; R_B = 3.04 %						

Supplementary Table 2. Structural parameters and reliability factors calculated from the synchrotron X-ray diffraction patterns for (a) disordered and (b) ordered Na_1RuO_3. The relatively high values of *B* for Na and O are explained by the weak stacking faults, which slightly distorts the local structure.

O-O in RuO_6	3 × 2.580(4) Å
	3 × 2.753(4) Å
	3 × 2.868(4) Å
	3 × 3.080(6) Å
Ru-O	3 × 1.937(4) Å
	3 × 2.043(3) Å
Ru-Ru	3 × 3.0320(1) Å
Na-O	3 × 2.301(5) Å
	3 × 2.481(5) Å
Na-Na	3 × 3.133(2) Å
Na-Ru	3.091(1) Å
Na-Vacancy	2.207(5) Å

Supplementary Table 3. Interatomic distances in O-Na_1RuO_3.
